# The association of serum phospholipids levels with chronic liver diseases: A systematic review of observational studies

**DOI:** 10.34172/hpp.025.43444

**Published:** 2025-07-15

**Authors:** Zahra Shahveghar Asl, Zohreh Ghoreishi, Faezeh Ghalichi, Meysam Zarezadeh, Alireza Ostadrahimi

**Affiliations:** ^1^Department of Clinical Nutrition, Faculty of Nutrition and Food Sciences, Tabriz University of Medical Sciences, Tabriz, Iran; ^2^Department of Nutrition and Food Sciences, Maragheh University of Medical Sciences, Maragheh, Iran; ^3^Student Research Committee, Tabriz University of Medical Sciences, Tabriz, Iran; ^4^Nutrition Research Center, Department of Clinical Nutrition, Faculty of Nutrition & Food Sciences, Tabriz university of Medical Sciences, Tabriz, Iran

**Keywords:** Liver diseases, Phospholipids, Systematic review

## Abstract

**Background:**

Chronic liver disease (CLD) influences the levels of diverse metabolites that may be related to its pathogenesis. The study aimed to indicate the relation between CLD and the levels of phospholipids.

**Methods:**

In this systematic review, PRISMA guidelines were considered for reporting the results. Up to November 2024, the databases of MEDLINE (through PubMed), Scopus, Web of Science, and Google Scholar were searched. Case-control (CC) and cross-sectional (CS) studies explored the link between CLD and serum phospholipids. The Newcastle-Ottawa scale (NOS) for CC studies and the modified NOS scale for CS studies were applied to evaluate the quality of the included articles.

**Results:**

A total of 11304 articles were included. Eleven thousand duplicates were removed, 9304 studies were excluded, and 343 full-text articles were reviewed. Fifteen CC studies and four CS studies were included in this study. Quality assessment using NOS revealed most studies had low to moderate risk of bias, with scores ranging from 4 to 8 out of 9.The included studies verified a significant association between the levels of total PL (TPL), phosphatidylcholine (PC), phosphatidylethanolamine (PE), phosphatidylserine (PS), phosphatidylinositol (PI), phosphatidic acid (PA), lysophosphatidylcholine (LPC), lysophosphatidylinositol (LPI) and lysophosphatidic acid (LPA) and liver diseases., with reported odds ratios ranging from 1.44 to 2.51 and correlation coefficients from -0.58 to 0.62.

**Conclusion:**

Phospholipid levels are associated with liver diseases. It is important to identify noninvasive ways to diagnose biological risk factors in patients with liver damage so they can be targeted for early treatment. Most of the included studies revealed significant alteration of phospholipid levels in CLD. Thus, the lipidome can predict liver dysfunction and prevent its attributed complications.

## Introduction

 Chronic diseases are a worldwide phenomenon that accounts for 80% of all deaths.^1^ Liver diseases are the most common diseases known around the world.^2^ Degeneration of liver tissue over time causes chronic liver disease (CLD).^3^ Non-alcoholic fatty liver disease (NAFLD) has been known to be the leading cause of CLD in developing countries.^4^ Other major risk factors of CLD are alcohol abuse, industrial toxins, diabetes, autoimmune diseases, malnutrition, use of certain drugs, and hepatotropic viruses.^5,6^ Because of the high incidence of complications of CLD, the quest for predictive and diagnostic biomarkers for CLD has received significant interest.^7^

 Phospholipids (PLs) are a class of lipids that consist of one molecule of alcohol, two molecules of fatty acids and one molecule of phosphate.^8^ Based on the alcohol group, several types of PLs existed, such as phosphatidylcholine (PC), lysophosphatidylcholine (LPC), phosphatidylethanolamine (PE), lysophosphatidylethanolamine (LPE), phosphatidylglycerol (PG), phosphatidylinositol (PI), or phosphatidylserine (PS).^9^ Different physiological and pathological states of the cells related to chronic diseases such as diabetes, kidney and liver diseases can lead to variations in PLs levels.^10-12^ Lower levels of PC and higher levels of PE have been shown in cases with NAFLD, non-alcoholic steatohepatitis (NASH), and simple steatosis (SS).^13^

 Previous studies reported that in CLD patients, the total plasma PLs were decreased.^14^ However, high concentrations of PC, sphingomyelin (SM), and low levels of LPC were related to hepatoma in participants with liver cirrhosis (LC).^15^ In a case-control (CC) study among children with NAFLD, an increase in PE and a decrease in PC, LPC, and LPE levels were observed.^16^ Another CC study indicated that the risk of NAFLD was related directly to plasma PL total saturated fatty acid (SFA) and C20:3n-6 levels, while the relationship between PL containing C22:6n-3 and the disease risk was the opposite.^17^ Moreover, SM (d18:1/24:0) was determined as a serum biological marker for liver injury in patients with hepatitis B virus.^18^ Based on mass spectrometry (MS) methods, a noninvasive diagnosis of NASH was defined based on a set of lipids and metabolites.^19^

 Major gaps exist in the knowledge about consistent use of PLs as diagnostic biomarkers for CLD in the clinical setting despite the following findings. Current studies also show PL-pattern discrepancies between various liver diseases, populations, and measurement methods, which restrain the PL application in early detection and prevention strategies. There also exists a lack of integrative syntheses evaluating the PL-level association with CLD subtypes, thus impeding the path toward standardized noninvasive diagnostic tools. From a health promotion perspective, identifying reliable biomarkers like PLs is critical for enabling early intervention, reducing disease progression, and alleviating the global burden of CLD. Early detection through noninvasive means can empower individuals and healthcare systems to implement lifestyle modifications, such as dietary interventions targeting lipid metabolism, and facilitate timely therapeutic strategies to prevent complications like cirrhosis and hepatocellular carcinoma. To date, no systematic review has comprehensively assessed the relationship between PL levels and liver diseases, underscoring the necessity of this study to fill this gap and inform health promotion initiatives aimed at improving liver health outcomes.

 This study contributes to identifying potential PL biomarkers that may enhance the detection and prevention of liver damage, supporting health promotion efforts to reduce CLD prevalence and its associated morbidity. By synthesizing evidence on PL alterations in CLD, this systematic review aims to provide a foundation for developing targeted screening programs and personalized interventions to promote liver health globally.

## Methods

###  Search strategy

 In the current systematic review study, the Preferred Reporting Items for Systematic Reviews and Meta-Analyses (PRISMA) guideline was followed (Supplementary file 1, Table S1).^20^ MEDLINE (PubMed), Web of Science, Scopus databases, and Google Scholar were searched to find all observational studies evaluating the association of PLs levels with liver diseases up to October 2022 without any date restriction. These searches were updated up to November 2024. The search strategies for each mentioned databases are shown in Table S2. To facilitate the review process and manage citations, articles were exported to Endnote software (Version X9; Thomson Reuters, Philadelphia, PA, USA).

###  Eligibility criteria 

 All original full-text English language articles that addressed the association between liver diseases and changes in levels of various PLs, including total PL (TPL), PC, PE, PS, PI, phosphatidic acid (PA), LPC, LPE, lysophosphatidic acid (LPA), lysophosphatidylinositol (LPI) and cephalin, were included in the present review. Studies were not included if they were intervention, cohort, review, or animal studies. Conference publications, book chapters, letters, editorials, posters, commentary, thesis, and the studies that their full-text versions were unavailable were excluded from the study.

###  Selection of the studies 

 Two independent reviewers systematically screened the articles. Controversies were resolved by discussion with the third reviewer. After removing duplicate articles, researchers checked the titles and abstracts according to the inclusion and exclusion criteria, and the full text of available articles was obtained. Then, the full text of the papers was further evaluated, and studies that could not meet the predefined criteria or had insufficient information were excluded. The CC and CS study designs were the main inclusion criteria for this study.

###  Data extraction

 Two reviewers, ZSH and FGH, extracted the related characteristics using a pre-developed data extraction sheet. The sheet comprised the first author’s name, year of publication, country, study population, sample size, gender, age, method of measurement and changes in the levels of various PLs. Also, the third reviewer was involved to recheck the extracted data, ensuring accuracy and consistency. In case of discrepancies or conflicts between the two initial reviewers extracted data, it is reasonable to conclude that these were resolved through discussion involving the third reviewer, similar to the process described for study selection.

###  Quality assessment

 The quality assessment of included CC articles was done using the Newcastle-Ottawa scale (NOS).^21^ NOS score range from 1 to 9, so the high-quality studies get higher scores. The risk of bias in the study is high if the participants receive five or fewer stars. The NOS scale contains three main sections: selection, comparability, outcome. The selection comprises four domains:

- Adequate definition of the case
- Representativeness of the cases
- Selection of controls
- Definition of controls.

According to the design or analysis, cases and controls were compared. Exposure consists of three parts: ascertainment of exposure, same ascertainment methods of cases and controls, and same non-response rate. Modified NOS was used to assess the quality of the included cross-sectional (CS) articles. The modified NOS scale contains three main sections: selection, comparability, and outcome. The selection comprises four domains: representativeness of the sample, sample size, ascertainment of exposure, and non-respondents. The items of comparability section are the comparability of subjects in different outcome groups based on the study design or analysis and controlling the confounding factors. The outcome comprised two sections: assessment of outcome and enough follow-up long for outcomes to occur.

## Results

###  Study selection

**Figure 1:**
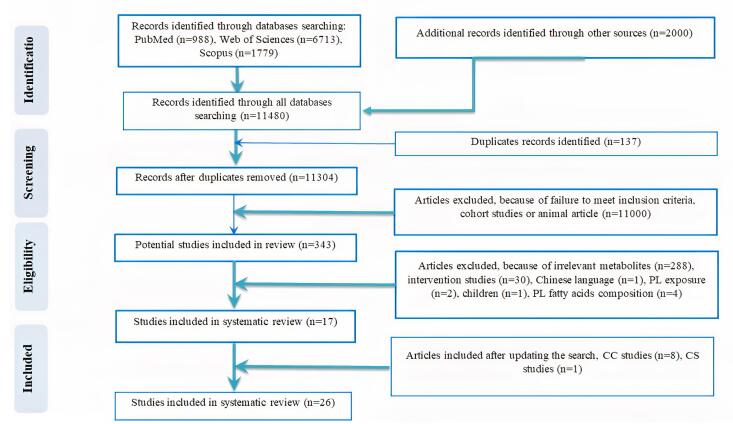
Flow diagram outlining included studies selection

 In the preliminary search, a total of 11480records were found based on an electronic database search of PubMed (n = 988), Web of Science (n = 6713), Scopus (n = 1779), and Google Scholar (n = 2000). After eliminating 137 duplicates, 11304 studies remained for further screening. Based on the title and abstract screening of the articles in the first stage, 11 000 articles were excluded because of being review, cohort, or animal studies. Then, the full text of the articles was critically assessed, of which 343 remained. Finally, according to the inclusion criteria, 17 studies were included in the current review. The search protocol was updated to extend the search period to November 2024. This update, conducted using the same search strategy and databases (Table S2), identified additional relevant studies, resulting in the inclusion of 8 more CC and 1 additional CS studies. In total, 21 CC and 5 CS studies were included in this review. The flow chart outlining the study selection process, including the updated search, is presented in Figure 1. All included articles reported at least one metabolite’s association with liver diseases. The flow chart outlining the selection of the studies is presented in Figure 1.

###  Study characteristics

 The articles included in the present review were published between 1966 and 2024. Study populations had liver complications, and both genders participated in most of the studies except for three, which did not report the exact participant number of males and females,^14,22,23^ and in one study, all participants were females.^24^ The sample size of the studies was from 15 to 600, and neither the follow-up periods nor the information on BMI was reported in any of the studies. The participants ‘s mean age was from 22.8 to 77.5 years, and only one study had not reported the mean age of the participants.^14^ There were ten studies performed in the China,^17,25-33^ two in Italy,^14,34^ Japan,^35,36^ Canada,^13,37^ and one each in Germany,^38^ United States,^22^ United Kingdom,^39^ India,^40^ Nigeria,^23^ Denmark,^41^ Norway,^42^ Brazil,^37^ Mexico^43^ and Wenzhou.^44^

 Participants had the following liver disorders: hepatitis,^23,25^ cholestatic jaundice,^23^ LC,^28,34,40,41^ hepatitis with LC,^27^ NAFLD,^13,17,29,35-37,43,44^, NASH,^13,33,36,38,44^ CLD,^14^ acute liver failure (ALF),^41^ hepatocellular carcinoma (HCC),^30^ metabolic dysfunction–associated steatotic liver disease (MASLD),^31^ hepatitis B virus-related acute-on-chronic liver failure (HBV-ACLF),^32^ primary biliary cholangitis (PBC)^24^ and drug-induced liver injury (DILI).^26^

 Measurement of PLs was done by thin layer chromatography (TLC) in eight included studies.^14,17,22,34,39,40,42^ Yamamoto et al^36^ used liquid chromatography mass spectrometry (LC–MS). Electrospray ionization mass spectrometry (ESI–MS) assay was performed to analysis lipidom in two studies.^22,38^ Ultra-high-performance liquid chromatography data combined with mass spectrometry (UPLC-MS) was applied in four studies.^25-27,29^ Other researchers performed gas liquid chromatography (GLC) and gas chromatography mass spectrometry (GC-MS),^26,35,41^ ultra-performance liquid chromatography coupled to electrospray ionization quadrupole time-of-flight mass spectrometry (UPLC-Q-TOF MS),^28,30,31^ high performance liquid chromatography–mass spectrometry/ mass spectrometry (HPLC–MS/MS),^32^ liquid chromatography coupled with high-resolution mass spectrometry (LC-HRMS),^24^ ultraperformance liquid chromatography - mass spectrometry/ mass spectrometry (UPLC−MS/MS),^33^ ultra-high performance liquid chromatography-mass spectrometry (UHPLC-QTOF/MS)^44^ and Nano electrospray infusion tandem mass spectrometry.^13^ Also, MS used to measure the metabolites.^43^ Only Ahaneku et al^23^ did not report the methodology of metabolite quantification. Characteristics of CC studies in this review are reported in Table 1. Table 2 presents the characteristics of included CS studies.

**Table 1:** Characteristics of included case-control studies and changes in levels of metabolites

**Authers**	**Country**	**Study population**	**Male/Female**	**Age (Case/control)**	**Method of Measurement **	**Outcome**
Cantoni et al 1975^14^	Italy	58 patients with CLD and 12 control subjects	NR	NR	TLC	Red cell: ↓ TPL, ↔PC, ↑LPC, ↓PE, ↓PS Plasma: ↓TPL, ↔PC, ↔LPC, ↔PE, ↔PS
Zheng et al 2012^17^	China	100 NAFLD patients and 100 healthy subjects	138/ 62	NAFLD: 44.97 ± 11.27/ Healthy:43.37 ± 12.24	TLC	↑TPL (SFA)
Ahaneku et al 1991^23^	Nigeria	13 patients with hepatitis and 11 patients with cholestatic jaundice and 20 healthy volunteers	NR^*^	Hepatitis: 32.00 ± 14.80, Cholestatic jaundice: 52.00 ± 17.70/ Healthy: 29.00 ± 06.90	NR	↑ TPL
de Oliveira et al 2024^24^	Brazil	30 PBC patients and 20 healthy controls	0/50	PBC: 38–82 Controls: 22–67	LC-HRMS	↓LPC(16:0), ↓LPC(16:1), ↓LPC(18:0), ↓LPC(18:1), ↓LPC (18:2), ↓LPC (20:4) ↓LPC (22:6), ↓PC (22:0) ↑LPC (18:1), ↑LPC (20:1), ↑LPC (20:5), ↑LPE (16:1), ↑PC (16:0/16:0), ↑PC (20:3/16:0), ↑PC (20:5/16:0)
Zhang et al 2017^25^	China	78 HBV patients and 19 Healthy volunteers	68/29	Group A: 56.40 ± 5.40, Group B: 56.70 ± 6.20, Group C: 55.30 ± 05.80, Group LC: 56.30 ± 5.30/ Healthy:57.00 ± 6.10	UPLC-MS	↓LPC (16:0), ↓LPC (18:0), ↓LPC (22:5)
Xie et al 2019^26^	China	56 DILI patients and 34 healthy controls	41/49	Non-severe group: 57.00 ± 14.00, Severe Group: 51.00 ± 15.00/ Healthy:51.00 ± 15.00	GC-MS and UPLC-MS	↓PC (22:6/16:0), ↓PC (16:1/18:2), ↓PC (18:0/20:3)
YE et al 2017^27^	China	83 HBV LC patients and 35 healthy individuals	62/56	Group A: 53.82 ± 8.12, group B:52.93 ± 9.24, group C: 57.19 ± 10.04/ Healthy:54.03 ± 7.53	UPLC-MS	↓LPC (16:0), ↓LPC (17:0), ↓LPC (18:0)
Huang et al 2013^28^	China	17 LC patients and 24 healthy individuals	28/31	LC: 51.71 ± 10.02/ Healthy:47.13 ± 8.08	UPLC-Q-TOF MS	↑LPC (16:0), ↑LPC (18:0), LPC (18:1), LPC (18:2)
Wang et al 2022^29^	China	149 NAFLD patients and 149 healthy controls	211/ 87	NAFLD: 48.41 ± 9.11 / Healthy:48.08 ± 10.83	UPLC-MS	↓LPC (24:1), ↓PC (19:1/0:0), ↑PC (14:1/16:1), ↑PS (16:0/18:0)
Lu et al 2015^30^	China	220 HCC patients and 224 normal controls	336/108	≤ 40 (n = 52) 41-50 (n = 123) 51-60 (n = 157) ≤ 60 (n = 112)	UPLC-Q-TOF MS	↓total PC, ↓total LPC, ↓total PE, ↓total LPE, ↓PE (18:0/0:0), ↓PC (16:1/2:0),
Shao et al 2024^31^	China	200 nonobese MASLD obese, 200 obese MASLD and 200 normal controls	430/170	nonobese MASLD obese: 40.7 ± 13.1/ obese MASLD: 41.0 ± 12.8/ Healthy:39.6 ± 10.0	UPLC-Q-TOF MS	↑PC, ↑PA, ↑PI, ↑PE
Wang et al 2017^32^	China	86 HBV-non-ACLF, 74 HBV-ACLF and 20 healthy controls	145/35	HBV-non-ACLF: 37.15 ± 7.98/ HBV-ACLF: 38.83 ± 6.38/ Healthy:31.54 ± 4.45	HPLC–MS/MS	↓LPC 22:6
Zhang et al 2024^33^	China	21 NASH and 30 healthy subjects	26/25	NASH: 37.9 ± 13.8 Healthy: 34.2 ± 4.3	UPLC−MS/MS	↓PC, ↓PS, ↓LPC, ↑PI
Yamamoto et al 2021^36^	Japan	31 patients with NAFLD (SS, n = 9; NASH, n = 27) and 8 healthy subjects	20/24	SS: 42.80 ± 16.40, NASH: 65.10 ± 14.00/ Healthy:22.80 ± 1.70	LC-MS	↓LPE (16:0), ↓LPE (18:0), ↓LPE (18:1), ↓LPE (18:2), ↓LPE (20:4), ↓LPE (20:5), ↓LPE (22:6), ↓PE (34:0), ↓PE (34:1), ↓PE (34:3), ↓PE (34:4), ↓PE 34:2 (16:0/18:2), ↓PE 36:1 (18:0/18:1), ↓PE 36:2 (18:0/18:2), ↓PE38:4(18:0/20:4), ↓PE 38:5 (18:1/20:4), ↓PE 40:6 (18:0/22:6)
Schwenger et al 2024^37^	Canada	83 NAFLD patients, 42 NASH patients and 30 NLO individuals	27/128	NAFLD: 49 [43, 57]^**^/ NASH: 50 [43, 57]/ NLO: 48 [38, 52]	LC–MS/MS	↓LPC (16:0), ↓LPC (17:0), ↓LPC (18:2)
Cairns et al 1983^39^	United Kingdom	16 chronic alcoholics and 5 non-insulin diabetic patients and 9 control patients	15/15	chronic alcoholics: 77.50/ diabetic patients: 54.20	TLC	↑ TPL
Vijayalakshmi et al 2006^40^	India	50 LC patients and 50 normal healthy volunteers	36/64	LC: 30-40/ Healthy:30-40	TLC	↓TPL
Clemmesen et al 2000^41^	Denmark	7 patients with LC and 10 ALF and 6 AOCLD and 11 healthy controls	18/15	LC: 49.00 ± 13.00, AOCLD: 48.00 ± 90.00, ALF: 42.00 ± 13.00/ Healthy: 31.00 ± 90.00	GLC	↓ TPL
Gjone et al 1966^42^	Norway	23 patients with liver diseases and18 normal humans	20/21	liver diseases: 56.19/ Healthy:28.70	TLC	↓TPL, ↔cephalin
Flores et al 2021^43^	Mexico	98 NAFLD cases and 100 healthy controls	50/148	NAFLD: 59.90 (57.80–62.00)/ Healthy: 61.50 (59.50–63.60)	MS	↓LPC (17:0), ↓LPC (15:0), ↓LPC (18:1),↓PC (17:0/18:1), ↓PC (17:0/18:2), ↔total PC, ↔total PE, ↔total LPE
Alvaro et al 1982^34^	Italy	8 patients with alcoholic LC and 7 healthy subjects	10/5	LC:33.00-59.00/ Healthy: 25.00-50.00	TLC	↓TPL, ↑PS, ↑PI, ↓PE, ↔PC

^*^NR, not reported; ^**^Median [IQR]. TPL, total phospholipid; LC, liver cirrhosis; TLC, Thin layer chromatography; PC, phosphatidylcholine; PE, phosphatidylethanolamine; PS, phosphatidylserine; PI, phosphatidylinositol; LPC, lysophosphatidylcholine; LPE, lysophosphatidylethanolamine; MASLD, metabolic dysfunction–associated steatotic liver disease; CLD, chronic liver disease; ALF, acute liver failure; AOCLD, acute on chronic liver disease; LC-MS, Liquid chromatography/mass spectrometry; LC-MS/MS, liquid chromatography- mass spectrometry/ mass spectrometry; GC-MS, gas chromatography mass spectrometry; UPLC-Q-TOF MS, ultra-performance liquid chromatography coupled to electrospray ionization quadrupole time-of-flight mass spectrometry; UPLC-MS, Ultra-high-performance liquid chromatography data combined with mass spectrometry; DILI, drug-induced liver injury; NAFLD, non-alcoholic fatty liver diseases; SS, simple steatosis; NASH, non-alcoholic steatohepatitis; HBV, hepatitis B virus; GLC, gas liquid chromatography; NLO, normal liver obese; HBV-non-ACLF, hepatitis B virus -non-related acute-on-chronic liver failure; HBV-ACLF, hepatitis B virus -related acute-on-chronic liver failure; HPLC–MS/MS, high performance liquid chromatography–mass spectrometry/ mass spectrometry; PBC, Primary Biliary Cholangitis; LC-HRMS, liquid chromatography coupled with high-resolution mass spectrometry; UPLC−MS/MS, ultraperformance liquid chromatography - mass spectrometry/mass spectrometry.

**Table 2:** Characteristics of cross-sectional studies and changes in levels of metabolites

**Authers**	**Country**	**Study population**	**Male/** **Female**	**Age**	**Method of measurement**	**Outcome**
Arendt et al 2013^13^	Canada	28 NAFLD patients and 9 healthy controls	20/17	SS: 40.90 ± 2.20, NASH: 42.80 ± 3.20, healthy: 40.40 ± 4.00	Nano electrospray infusion tandem mass spectrometry	Hepatic: ↓PC/PE ratio, ↓PC,­ ↑PE, erythrocytes: ↓PC/PE ratio, ↔PE, ↓PC
Xiao et al 2001^22^	United States	15 patients with ovarian cancer and 15 patients with benign liver diseases	NR	ovarian cancer: 48.00–86.00 benign liver diseases: 43.00–74.00	TLC, ESI-MS	↑­acyl-LPA, ­↑alkyl-LPA, ↑alkenyl-LPA, ↑­LPI (16:0), ↑­LPI (18:0), ↔TPL, ↑ ­LPC (17:0), ↑­LPC (6:0), ↑­LPC (8:0), ­↑LPC (10:0),­ ↑LPC (12:0), ↑­LPC (14:0), ↑LPC (16:0),­ ↑LPC (18:0), ↑­LPC (20:0), ↑­LPC (22:0), ↑­LPC (24:0)
Ogawa et al 2020^35^	Japan	83 NAFLD patients non-ballooning and 49 NAFLD patients with ballooning	65/67	patients non-ballooning: 49 (17–76) patients with ballooning: 59 (24–79)	GC/MS	↑­PC, ↓LPC, ↓LPE
Krautbauer et al 2016^38^	Germany	6 patients with fatty liver and 4 with NASH and 11 subjects without fatty liver	21/0	63.00 (47.00 – 84.00)	ESI–MS	PC:­ ↑PC (SFA), ↓PC (PUFA), LPC:↔total LPC,↔ LPC (SFA), ↓LPC (PUFA), ↑­LPC (16:1), ­ ↑LPC (20:3), ↓LPC (18:2), ↓LPC (20:4), ↓LPC (22:6), PE: ↓total PE, ­↑PE (SFA), ↓PE (PUFA), PI: ↓total PI, ↓PI (38:4), ↓PI (PUFA), PS: ↓Total PS, ↓PS (SFA), ↓PS (PUFA), ↓PS (36:1), ↓PS (40:6)
Wang et al 2021^44^	Wenzhou	30 NAFLD patients and 10 cases of NASH	21/9	NAFL: 47.25 ± 11.04, NASH: 44.50 ± 10.92	UHPLC-QTOF/MS	↑PC (22:0/18:1),­ ↑PE (18:0/22:5), ­↑PC (O-22:2/12:0), ↑­PC (26:1/11:0), ↓PC (22:6/0:0),↓PC (16:1/0:0),

NR, not reported; TPL, total phospholipid; LC, liver cirrhosis; TLC, Thin layer chromatography; TPL, total phospholipid; PC, phosphatidylcholine; PE, phosphatidylethanolamine; PS, phosphatidylserine; PI, phosphatidylinositol; LPA, lysophosphatidic acid; LPC, lysophosphatidylcholine; LPI, lysophosphatidylinositol; NAFLD, non-alcoholic fatty liver diseases; SS, simple steatosis; NASH, non-alcoholic steatohepatitis; ESI–MS, electrospray ionization mass spectrometry; PUFA, polyunsaturated fatty acid; SFA, saturated fatty acid; UHPLC-QTOF/MS, ultra-high performance liquid chromatography-mass spectrometry.; GC/MS, gas chromatography–mass spectrometry.

###  Quality assessment

Tables 3 and 4 show quality assessment tools for CC and CS studies, respectively. Decisions about the risk of bias for each item are shown as scores of stars across included CC and CS studies. Of note, a minor risk of bias exists based on the results of the included studies.

**Table 3:** Risk of bias indicating case control studies’ quality assessment at an individual level

**Study**	**Selection**				**Comparability**		**Exposure**			**Total**
	**Adequate definition of the case**	**Representativeness of the cases**	**Selection of controls**	**Definition of controls**	**Comparability of cases and controls**		**Ascertainment of exposure**	**Cases and controls: same ascertainment method**	**Cases and controls: same non-response rate**	
					**Design**	**Analysis**				
Cantoni et al 1975^14^	*	*	*	*	*	*	*	-	-	7/9
Zheng et al 2012^17^	*	*	*	*	-	-	*	-	-	5/9
Ahaneku et al 1991^23^	*	*	-	*	-	-	*	*	*	6/9
de Oliveira et al 2024^24^	*	*	*	*	*	*	*	*	-	8/9
Zhang et al 2017^25^	*	*	-	*	-	-	*	*	*	6/9
Xie et al 2019^26^	*	*	*	-	-	-	*	*	*	6/9
YE et al 2017^27^	*	*	-	*	-	-	*	*	-	5/9
Huang et al 2013^28^	*	*	*	*	*	*	*	*	-	8/9
Wang et al 2022^29^	*	*	-	*	*	-	*	*	-	6/9
Lu et al 2015^30^	*	*	*	*	*	*	*	*	-	8/9
Shao et al 2024^31^	*	*	*	*	-	*	*	*	-	7/9
Wang et al 2017^32^	*	*	*	-	*	*	*	*	-	8/9
Zhang et al 2024^33^	*	*	*	-	*	*	*	-	-	6/9
Yamamoto et al 2021^36^	*	*	*	*	-	-	*	-	-	5/9
Schwenger et al 2024^37^	*	*	-	*	*	*	*	-	-	6/9
Cairns et al 1983^39^	*	*	-	*	*	*	*	*	-	7/9
Vijayalakshmi et al 2006^40^	*	*	-	*	*	*	*	*	*	8/9
Clemmesen et al 2000^41^	*	*	*	*	-	-	*	-	*	6/9
Gjone et al 1966^42^	*	*	-	*	-	-	*	-	*	5/9
Flores et al 2021^43^	*	*	*	-	*	*	*	*	-	7/9
Alvaro et al 1982^34^	*	*	*	*	-	-	*	-	-	5/9

* = low risk; - = high risk.

**Table 4:** Risk of bias indicating cross sectional studies’ quality assessment at an individual level.

**Study**	**Selection**				**Comparability**		**Outcome**		**Total**
	**Representativeness of the sample**	**Sample size**	**Ascertainment of exposure**	**Non-respondents**	**Design**	**Analysis**	**Assessment of outcome**	**Adequacy of follow up**	
Arendt et al 2013^13^	*	-	*	*	*	*	*	*	7/8
Xiao et al 2001^22^	*	-	-	*	*	*	*	-	5/8
Ogawa et al 2020^35^	*	*	-	-	*	*	*	-	5/8
Krautbauer et al 2016^38^	*	-	-	-	*	*	*	-	4/8
Wang et al 2021^44^	*	-	-	-	*	*	*	-	4/8

* = low risk; - = high risk

###  Association of metabolites with the liver diseases

Table 1 reports changes in different PLs in CC studies. Table 2 shows changes in the lipidome in CS studies.

####  Total phospholipid (TPL)

 Eight CC studies measured TPL levels. In three CC studies, the levels of TPL increased,^17,23,39^ while five studies showed a decrease in TPL levels in the patient group compared to the control.^14,34,40-42^ In a CS study, Xiao et al^22^ found no statistically significant difference in TPL levels in ascites samples from subjects with ovarian cancer to those with non-malignant liver disorders.

 Total phospholipid (SFA) positively associated with the risk of NAFLD (OR = 1.44, 95 %CI = 1.11–1.88).^17^ Among cholestatic jaundice patients, alanine amino-transferase correlated positively with TPL (r = 0.623, *P* < 0.05).^23^ In red blood cells, a negative correlation (r = -0.4906, *P* < 0.05) was observed between the cholesterol: TPL ratio and the percent PE within TPL.^34^ Based on the Child-Pugh system, the levels of platelet components (platelet count, cholesterol/TPL ratio, and total ATPases) in patients with liver cirrhosis were highly correlated with the degree of liver damage; Class A (rs = -0.4, *P* < 0.05), Class B (rs = -0.72, *P* < 0.02), Class C (rs = -0.54, *P* < 0.01).^40^

####  Phosphatidylcholine (PC) 

 Phosphatidylcholine levels were measured in nine CC studies.^14,24,26,29-31,33,34,43^ In two studies,^14,34^ there were no significant differences in PC levels between the control and liver disease, and in four studies,^26,29,30,33^ several PCs were significantly lower in cases compared to controls. Also, in two CC studies PC levels were higher than controls.^24,31^ Flores et al^43^ explored that levels of PC (17:0/18:1) and PC (17:0/18:2) were reduced, whereas total PC levels were not different. Among the CS studies, four studies measured levels of PC. Phosphatidylcholine levels were decreased in one study.^13^ Two studies^38,44^ found that some species of PCs increased, while other PCs such as PC (SFA), PC polyunsaturated fatty acid (PUFA), PC (22:6/0:0), and PC (16:1/0:0) decreased across the progress of liver damage. Another CS study reported that PC levels in the patient group were raised.^35^

 Strong positive correlations between PCs and TAGs, CEs, CERs, LPCs, and SMs were reported by Flores et al.^43^ The lipid species in each of the subclasses had strong positive correlations with each other. Phosphatidylcholine (19:1) had a negative correlation (*P* < 0.05, rho = −0.473) with waist circumference, glutamyl transpeptidase, and serum levels of triglycerides.^29^ Krautbauer et al^38^ noted a positive association between PC 38:6 (r = 0.525, *P* = 0.015) in tumor tissue (r = 0.472, *P* = 0.031) with the serum levels. The PC/PE ratios in the erythrocytes and liver showed no significant correlation in patients and controls combined (Spearman’s r = 0.276, *P* = 0.203).^13^ A positive correlation exists between PC (14:0/18:2) and NAFLD activity score (NAS) (regression values: 0.43, *P* = 0.01). In contrast, a negative correlation between NAS and PCs (saturated or monounsaturated), such as PC (22:6/0:0), PC (20:4/0:0), and PC (16:1/0:0) (regression value: -0.56, -0.58 and -0.56, *P* = 0.001, 0.0006 and 0.001, respectively). The liver fibrosis score and PC (18:0/0:0) showed a positive correlation (regression value: 0.36, *P* = 0.04). Whereas PC (O-22:2/16:1) and PC (O-22:0/0:0) had a negative correlation with liver fibrosis scores (regression values: -0.45 and -0.41, *P* = 0.01 and 0.02, respectively).^44^

####  Phosphatidylethanolamine (PE) 

 In seven CC studies,^14,30,31,34,42,43,36^ PE levels were measured. In three studies, PE levels were significantly lower in patients compared to normal ones.^30,34,36^ A decline in PE levels was observed in a CC study.^31^ Cantoni et al^14^ reported reduced red blood cells’ PE concentration and no change in plasma levels in patients with CLD. There was no total PE level difference between patients and healthy people in another CC study.^43^ Three CS studies^13,38,44^ measured PE levels. Levels of PE were increased in one CS study.^44^ In another study, researchers observed that some types of PEs were increased while some other types (alkyl/alkenyl-PE (PE[O])) were decreased.^38^ Another CS study^13^ reported that the PE levels in erythrocytes were similar in patients and healthy controls. Cephalin is the common name for phosphatidylethanolamine. Gjone et al^42^ showed that the cephalin concentrations were similar between patients with liver disease and healthy participants.

 The percent PE within red cell phospholipids and the cholesterol: TPL molar ratio were shown to be negatively correlated (r = -0.4906, *P* < 0.05).^36^ Positive correlations existed between lipid species across the subclasses.^43^ The PC/PE ratios in the erythrocytes and liver of both patients and controls did not significantly correlate (Spearman’s r = 0.276, *P* = 0.203).^13^ Phosphatidylethanolamine species and T-staging were not associated. There was no correlation between PE level changes in the tumor and systemic levels.^38^ Positive correlations existed between PE (18:0/22:5) and PC (14:0/18:2) levels with NAS (regression values: 0.46 and 0.43, *P* = 0.01, respectively).^44^

####  Phosphatidylserine (PS)

 The PS levels were measured in four CC studies.^14,29,34^ Serum PS levels were significantly higher in NAFLD patients than in healthy individuals in two CC studies.^29,34^ Cantoni et al^14^ observed that PS levels in the liver disease and control groups did not significantly differ. Reduced PS levels were reported in other CC studies.^33^ One CS study^38^ measured PS levels. Phosphatidylserine levels were reduced in the patients’ group and the p53 ratio negatively correlated with PS 36:1 (r = - 0.586, *P* = 0.005). Also, PS levels in the tumor were not associated with systemic levels.

####  Phosphatidylinositol (PI)

 Three CC studies measured PI levels. Significantly high PI levels in patients compared to healthy ones were reported in these studies.^31,33,34^ One CS study^38^ reported that PI levels were reduced. Phosphatidylinositol species were not related to T-staging. The PI 38:4 levels and the p53 ratio did not correlate. Systemic levels of PI did not correlate with PI levels in the tumor. There was a clear association between PI and moderate to severe steatosis in patients (OR: 2.51; 95% CI: 1.93–4.81).^31^

####  Lysophosphatidylcholine (LPC)

 In eleven CC studies,^14,24,25,27-30,32,33,37,43^ LPC levels were measured. As seen in Table 1, eight studies reported decreased LPC.^25,27,29,30,32,33,37,43^ In contrast, Huang et al^28^ showed increased levels of LPC. In other CC studies, the levels for LPC in liver disease and control did not differ significantly from one another.^14^ Three CS studies measured LPC levels.^22,35,38^ One study showed increased LPC levels in malignant ascites compared to nonmalignant ascites.^22^ In contrast, another CS study showed a decline in LPC levels among the patients compared to the controls.^35^ Moreover, Krautbauer et al^38^ demonstrated increased levels of LPC (16:1) and LPC (20:3) and decreased levels of LPC (PUFA), LPC (18:2), LPC (20:4) and LPC (22:6) and without any changes in total LPC and SFA LPC.

 There was a correlation between LPC (16:0), LPC (18:0), and LPC (22:5) and HCC differentiation grades.^25^ A correlation was found between changes in LPC levels (16:0, 17:0, 18:0, 18:1, 18:3, 18:1, 20:1, 20:3) with Child-Pugh score.^27^ BMI, glucose, and triglycerides negatively correlated with LPC (24:0).^29^ Most LPCs, PCs, and SMs positively correlated with CEs and CERs. Furthermore, there was a positive correlation between PCs with SMs and LPCs. Positive correlations were observed between lipid species across the subclasses.^43^ In HCC tissues, changes in LPC 22:6 levels were correlated with serum levels. There was no relation between T-staging and any of the specific LPC species. Serum levels of LPC (22:6) and LPC (22:6) in tumor tissue had a positive association (r = 0.472, p = 0.031).^38^ Lysophosphatidylcholine (LPC)16:0 and LPC (18:0) showed a negative correlation with the Model for End-Stage Liver Disease (MELD) score. There is also a significant relationship between LPC (16:0) and LPC (18:0) (*P* < 0.0001; r = 0.942). Similarly, a positive correlation between PC (32:0) and ALT was reported (*P* = 0.0003; r = 0.431).^32^

####  Lysophosphatidylethanolamine (LPE)

 Four CC studies reported LPE levels.^24,30,43,36^ In two studies, the patients’ LPE levels significantly reduced compared to the healthy volunteers, except LPE 18:0.^30,36^ Another study detected no difference in total LPE levels between patients with NAFLD and healthy ones.^43^ The R2 values for all of the LPE analyses were more than 0.9972. The LPE subclasses showed significant positive correlations with each other.^43^ Increased LPE levels were shown in patients compared to the controls in a CC study.^24^ Only one CS study reported a decrease in LPE levels.^35^

####  Lysophosphatidic acid (LPA) and Lysophosphatidylinositol (LPI)

 In one CS study conducted by Xiao et al,^22^ the levels ofLPA types and LPI were increased in ovarian cancer patients compared with subjects with benign liver diseases.

####  Phosphatidic acid (PA)

 Only one CC study indicated increased PA levels in MASLD obese patients compared to the controls.^35^

## Discussion

 The current systematic review summarized twenty-one CC and five CS studies. The findings showed that all CC and CS studies reported significant correlations between serum TPL, PC, PE, PS, PI, PA, LPC, and LPE with CLD.

 The liver is the main organ responsible for cholesterol, PL, triglyceride, and lipoprotein metabolism. The functional damage of the liver would lead to the decreased ability to synthesize many vital biomolecules, including lipids.^40^ Recent studies have shown a significant association between alteration in plasma PL pattern and the pathogenesis of CLD.^45,46^ Changes in the lipid composition of plasma lead to structural changes and transfer of PL from plasma to the liver, which disables PL biosynthesis and accelerates the severity of liver diseases.^14,46^ The dietary fat composition modifies gene transcription and signal transduction^13^ and affects membrane function, cell proliferation, and apoptosis, by regulating hepatic lipid metabolism.^38^

 The alterations in the PL pattern in CLD can be justified in several ways. Abnormal fatty acid composition is caused by impaired fatty acid metabolism (beta-oxidation or intracellular fatty acid transport).^41^ In LC patients, defects in the conversion of linolenic acid to arachidonic acid^34^ increase Δ-9 desaturase and reduce elongase activity.^47^ Thus, changes in the fatty acid levels can lead to various PL patterns. Furthermore, reduction in biliary secretion of certain PLs,^48^ malnutrition, malabsorption, pancreatic insufficiency,^49^ dysfunction of pancreatic exocrine secretion,^13^ reduced secretion of phospholipase A2, imbalance of gut microflora,^50^ and the presence of lecithinase-positive bacteria in gut microflora^51^ contribute to changes in PLs pattern in CLD. In addition, the deterioration of the activity of enzymes such as lecithin cholesterol acyltransferase, phospholipases, hepatic lipase, endothelial lipase, and LPC acyltransferases alter lipid metabolism in LC patients.

 Ahaneku et al^23^ reported a significant correlation between TPL, HDL-phospholipid, and phospholipid-to-cholesterol ratio levels in cholestatic jaundice and acute viral hepatitis. The increase in HDL-phospholipid levels could be due to the rise in primary apolipoprotein E-rich HDL (LpE) observed in biliary obstruction. Cairns et al^39^ indicated PLs alteration in the liver of diabetic and cirrhotic subjects does not mainly reflect the free fatty acids, dietary deficiency, or malabsorption of PUFAs; instead, changes in PL arrangement and decreased hepatocyte membrane fluidity. The cellulose response to hormones and drugs during cell division, and regeneration may also explain the relative decrease in the fluidity of the hepatocyte membrane. Zheng et al^17^ showed that plasma PL levels, total SFA, and C20:3n-6 had a positive association with the risk of obtaining NAFLD, and high plasma concentrations of PL C22:6n-3 had a negative relationship with the risk of obtaining NAFLD. The decline in delta-5 desaturase activity among patients with NAFLD may be related to the high levels of C20:3n-6. Additionally, in agreement with previous studies, plasma PL fatty acid does not reflect actual dietary intake; it is the primary biomarker for investigating the association between fatty acid intake and disease risk. Cantoni et al^14^ reported that changes in plasma PLs may exhibit hepatocyte lipids disruption. However, liver cell PLs are significantly associated with those in the erythrocytes than the plasma. Alterations in plasma enzyme lecithin acyltransferase (LCAT), reduction in the biosynthesis of PL in liver diseases, alteration in the transformation of PL from plasma to the erythrocytes, enhanced bile salt concentrations in liver disorders, and inability to synthesize PLs for regenerating hepatocytes have been associated with changes in red blood cell PLs composition. The principal changes in PL pattern in severe and prolonged cases of parenchymatous and biliary duct obstructive jaundice liver diseases are due to the partial impairment of lysolecithin.^42^ In cirrhosis patients with hepatocellular carcinoma, plasma PL levels show a significant negative relationship with total bilirubin and alkaline phosphatase, explaining the increased levels of alkaline phosphatase and decreased levels of PLs.^40^

 It has been mentioned that during hepatocyte injury, phosphatidylethanolamine-N-methyltransferase (PEMT) activity declines, which increases the disease severity and leads to reduced synthesis of PC.^26^ Subjects detected with PEMT functional single nucleotide polymorphism are prone to developing NAFLD.^52^ Phosphatidylcholine may also be depleted through adaptation to SM and diacylglycerol production.^53^ Ether-phospholipids are the byproducts of the liver. These changes may be due to the reduced dietary consumption of choline and ethanolamine during steatosis, as they are important for the synthesizing of ether-phospholipid.^16^ Low amounts of dietary choline elevate hepatocarcinogenesis and carcinogens such as diethylnitrosamine in animal models.^54,55^ On the other hand, diets supplemented with PC defend from acquiring HCC, partially by augmenting cellular apoptosis.^56^ Lysophosphatidylcholine species 14:0, 20:3, and 22:6 are significantly altered in HCC plasma in comparison to cirrhotic patients.^38^

 The reduced levels of lysolecithin in parenchymatous liver diseases reflect reduced synthesis. Low plasma lysolecithin may also be due to increased breakdown or acylation of lecithin. Increased acylation may occur in conditions with increased plasma-free fatty acid levels. This may explain the low plasma lysolecithin values observed in abnormal states.^42^

 Arendt et al^13^ reported that PL metabolism and diet affect the PC to PE ratio, PC, and PE levels. PC homeostasis is based on choline obtained from diet and the hepatic conversion of PE to PC. These paths are controlled via interconnecting the metabolism of methionine and folate. Suboptimal choline, betaine, methionine, or folate intake could lead to reversible hepatic steatosis and apoptosis of hepatocytes. The lower PC/PE ratio may be due to decreased PEMT activity, which catalyzes the hepatic transformation of PE to PC by the PEMT gene mutation. This could indirectly result in the enhancement of PE substrates and/or more usage of PE in the membrane by hepatic cells for PC loss.^13^

 Cantoni et al^14^ reported that the red blood cell’s various plasma environment, due to the high bile salt content of liver diseases, is responsible for the reduced biosynthesis of PLs in liver diseases and may alter the transformation of the PL to the red blood cell from plasma.^14^ Krautbauer et al^38^ indicated that that several lipid species are altered during cancer in the liver, which could be responsible for tumor growth and survival. In this context, MUFA and PUFA PS species are markedly repressed in tumors, emphasizing their role in tumor growth.

 The PI determination in the membrane of erythrocytes is usually ignored due to the insufficient amounts in regular settings and the complications related to separating acidic PLs such as PS and PI.^34,39^ Alvaro et al^34^ recommend that the different lipid configurations of red blood cell plasma membranes may correlate with membrane PL circulation variations. Reduced PI levels may be due to mutations in the TP53-gene, which is related to decreased PI levels (38:4) and augmented PI levels (36:1). According to research, in HCC tissues, PI (38:4) and PI (36:2) declined significantly.^38^

 In normal human circulation, LPC is the primary bioactive plasma lipid and the most abundant cellular PL, mostly correlated with albumin and lipoproteins.^27,57^ Lysophosphatidylcholine is produced by the phospholipase A2 action on membrane PC.^57^ In fact, LCAT is responsible for catalyzing the transacylation of the PC fatty acid residues into free cholesterol. Subsequently, it results in the formation of LPC and cholesterol esters. Ye et al^27^ indicate that reduced LPC levels could be considered a precise index for assessing dietary conditions in hepatitis B cirrhosis. In contrast, Huang et al^28^ revealed a meaningful augment in fecal LPC levels among patients with cirrhosis. Fecal LPC mainly originates from phospholipase A2 or the gut microflora hydrolysis of PC, both dietary and biliary. LPC is the main constituent of dietary and biliary PL. In cirrhotic patients, the biliary PL secretion excretion is reduced.^58^ High levels of fecal LPC could be due to pancreatic, which inadequacy is supposed to be because of elevated LPC excretion in cystic fibrosis patients. Also, in these patients, pancreatic exocrine function is disrupted, which reduces phospholipase A2 secretion and decreases PC digestion and absorption. The phospholipase A2 secretion impairment causes increased fecal LPC excretion in cirrhotic patients.^59^ Moreover, lecithinase-positive bacteria in the gut microflora are dietary-dependent. In addition, the elevated fecal LPC may be because of the gut microflora imbalance acquired during cirrhosis.^60^

 The causes of decreased LPE levels were the high activity of lysophosphatidylcholine acyltransferase (LPCAT 3,4), lysophosphatidylethanolamine acyltransferase 1 (LPEAT 1), and reduced levels of PE containing linolenic acid, arachidonic acid, and docosahexaenoic acid. Inflammation leads to a rise in LPCAT3, 4, and LPEAT 1 in the liver.^61^ Tanaka et al^62^ reported that high levels of inflammatory cytokines, tumor necrosis factor-α, and transforming growth factor-ß1 induce elevated levels of LPCAT 1, 2, 3, and 4 and decline LPC. Furthermore, PE comprises linolenic acid, arachidonic acid, and docosahexaenoic acid. Hence, it is possible that oxidative stress highly induces PE degradation.^63^ Also, since PE biosynthesis occurs in the endoplasmic reticulum,^64^ endoplasmic reticulum stress induced in NAFLD patients may lead to lower levels of LPE.^65^

 In Xiao et al’s study, the elevated levels of LPL content may not be due to the generalized overproduction of PLs.^22^ Lysophosphatidic acid stimulates tumor cell proliferation, and massive amounts of bioactive lipids play a major role in the development and metastasis of tumors. In fact, LPIs and total alkyl- and alkenyl-LPAs are significant determinants for distinguishing malignant from nonmalignant ascites.^22^

 Ten of the 26 articles reported incomplete results from the design and analysis. Some studies needed to explain the selection and definition of the control group fully. The eight studies should have mentioned the ascertainment method for the case and control groups. In most case-control studies, the nonresponse rate was not the same for the case and control groups. In four of the five cross-sectional studies included in this study, the exact calculation of the sample size should have been mentioned. Also, most studies did not say the ascertainment of exposure.

 The strength of the present review was the high number of included studies, which made it possible to generalize the results. Furthermore, the quality of the studies was assessed based on the NOS, and most studies were demonstrated as high-quality. Nevertheless, this review had some limitations. Due to the studies’ heterogeneity, especially in outcomes and methods of analysis, conducting a meta-analysis was avoided. Omitting non-English studies that may have added language bias and also the various methods reported for measuring PLs were other constraints of the study. Also, the authors did not register their systematic review protocol with an international registry.

## Conclusion

 This systematic review revealed that the PLs levels are possibly associated with various chronic liver dysfunctions. Most of the included studies demonstrated that the levels of PLs were different in patients with CLD in comparison to healthy individuals. The findings suggest that serum levels of PLs as a diagnostic biomarker that may help to improve the detection and prevention of biological liver disorders.

## Competing Interests

 The authors have no relevant financial or non-financial interests to disclose.

## Data Availability Statement

 The authors confirm that the data supporting the findings of this study are available within the article [and/or] its supplementary materials.

## Ethical Approval

 Ethic declaration is in accordance with the Declaration of Helsinki. Ethical approval for this study was obtained from ethics committee of Tabriz University of Medical Sciences (ethics number: IR.TBZMED.REC.1400.1102).

## Supplementary Files

Supplementary file 1: The PRISMA guideline is shown in Table S1 and the search strategies for databases are shown in Table S2.
