# Supplementary material for: The association of serum phospholipids levels with chronic liver diseases: A systematic review of observational studies
Source: Health Promot Perspect. 2025 Jul 15;15(2):110–21. doi: 10.34172/hpp.025.43444 (PMC12450576; doi:10.34172/hpp.025.43444)
Supplement: Supplementary file 1 — The PRISMA guideline is shown in Table S1 and the search strategies for databases are shown in Table S2. [file hpp-15-110-s001.pdf]

Shahveghar Asl et al, **Health Promotion Perspectives.** 2025;15(2):110-S1
doi: 10.34172/hpp.025.43444
https://hpp.tbzmed.ac.ir

Supplementary Table 1. PRISMA checklist; the association of serum phospholipids levels with chronic liver diseases: A systematic review of observational studies

Section/topic	#	Checklist item	Reported on page #
**TITLE**			
Title	1	Identify the report as a systematic review, meta-analysis, or both.	Title page
**ABSTRACT**			
Structured summary	2	Provide a structured summary including, as applicable: background; objectives; data sources; study eligibility criteria, participants, and interventions; study appraisal and synthesis methods; results; limitations; conclusions and implications of key findings; systematic review registration number.	1
**INTRODUCTION**			
Rationale	3	Describe the rationale for the review in the context of what is already known.	2
Objectives	4	Provide an explicit statement of questions being addressed with reference to participants, interventions, comparisons, outcomes, and study design (PICOS).	2
**METHODS**			
Protocol and registration	5	Indicate if a review protocol exists, if and where it can be accessed (e.g., Web address), and, if available, provide registration information including registration number.	-
Eligibility criteria	6	Specify study characteristics (e.g., PICOS, length of follow-up) and report characteristics (e.g., years considered, language, publication status) used as criteria for eligibility, giving rationale.	3
Information sources	7	Describe all information sources (e.g., databases with dates of coverage, contact with study authors to identify additional studies) in the search and date last searched.	3-4
Search	8	Present full electronic search strategy for at least one database, including any limits used, such that it could be repeated.	3 and Supplementary Table 2
Study selection	9	State the process for selecting studies (i.e., screening, eligibility, included in systematic review, and, if applicable, included in the meta-analysis).	6
Data collection process	10	Describe method of data extraction from reports (e.g., piloted forms, independently, in duplicate) and any processes for obtaining and confirming data from investigators.	6
Data items	11	List and define all variables for which data were sought (e.g., PICOS, funding sources) and any assumptions and simplifications made.	6,Tables 1 and 2
**Section/topic**	**#**	**Checklist item**	**Reported on page #**
Risk of bias in individual studies	12	Describe methods used for assessing risk of bias of individual studies (including specification of whether this was done at the study or outcome level), and how this information is to be used in any data synthesis.	4
Summary	13	State the principal summary measures (e.g., risk ratio, difference in means).	-

measures			
Synthesis of results	14	Describe the methods of handling data and combining results of studies, if done, including measures of consistency (e.g., I^2) for each meta-analysis.	-
Risk of bias across studies	15	Specify any assessment of risk of bias that may affect the cumulative evidence (e.g., publication bias, selective reporting within studies).	4
Additional analyses	16	Describe methods of additional analyses (e.g., sensitivity or subgroup analyses, meta-regression), if done, indicating which were pre-specified.	-
**RESULTS**			
Study selection	17	Give numbers of studies screened, assessed for eligibility, and included in the review, with reasons for exclusions at each stage, ideally with a flow diagram.	5, Figure 1
Study characteristics	18	For each study, present characteristics for which data were extracted (e.g., study size, PICOS, follow-up period) and provide the citations.	5, Tables 1-2
Risk of bias within studies	19	Present data on risk of bias of each study and, if available, any outcome level assessment (see item 12).	6, Figure 2,3
Results of individual studies	20	For all outcomes considered (benefits or harms), present, for each study: (a) simple summary data for each intervention group (b) effect estimates and confidence intervals, ideally with a forest plot.	6-8, Tables 1-2
Synthesis of results	21	Present results of each meta-analysis done, including confidence intervals and measures of consistency.	-
Risk of bias across studies	22	Present results of any assessment of risk of bias across studies (see Item 15).	6
Additional analysis	23	Give results of additional analyses, if done (e.g., sensitivity or subgroup analyses, meta-regression [see Item 16]).	-
**DISCUSSION**			
Summary of evidence	24	Summarize the main findings including the strength of evidence for each main outcome; consider their relevance to key groups (e.g., healthcare providers, users, and policy makers).	8-11
Limitations	25	Discuss limitations at study and outcome level (e.g., risk of bias), and at review-level (e.g., incomplete retrieval of identified research, reporting bias).	11
Conclusions	26	Provide a general interpretation of the results in the context of other evidence, and implications for future research.	11
**FUNDING**			
Funding	27	Describe sources of funding for the systematic review and other support (e.g., supply of data); role of funders for the systematic review.	11

Supplementary Table 2. The association of serum phospholipids levels with chronic liver disease: A systematic review. Method of the database search strategy using PubMed, Scopus, and Google Scholar

Database (Search conducted up to September 2022)	Search terms *	Number of studies searched

PubMed	((("phospholipids"[MeSH Terms] OR "phospholipid"[Title/Abstract] OR "Glycerophospholipid"[Title/Abstract] OR "Glycerylphosphorylcholine"[Title/Abstract] OR "phosphatidylcholine"[Title/Abstract] OR "phosphatidylcholines"[MeSH Terms] OR "12"[All Fields]) AND "dipalmitoylphosphatidylcholine"[Title/Abstract]) OR "Dimyristoylphosphatidylcholine"[Title/Abstract] OR "Lecithins"[Title/Abstract] OR "glycerol"[Title/Abstract] OR "Phosphatidylethanolamines"[Title/Abstract] OR "Phosphatidylglycerols"[Title/Abstract] OR "Phosphatidylinositols"[Title/Abstract] OR "Phosphatidylserines"[Title/Abstract] OR "serum phospholipid"[Title/Abstract] OR "Lysophosphatidylcholine"[Title/Abstract]) AND ("fatty liver"[Title/Abstract] OR "non alcoholic fatty liver disease"[Title/Abstract] OR "liver failure"[Title/Abstract] OR "liver cirrhosis"[Title/Abstract] OR "Hepatitis"[Title/Abstract] OR "alanine transaminase"[MeSH Terms] OR "aspartate aminotransferases"[MeSH Terms] OR "alkaline phosphatase"[MeSH Terms] OR "gamma-Glutamyltransferase"[MeSH Terms] OR ("non-alcoholic"[All Fields] AND "non alcoholic fatty liver disease"[MeSH Terms]) OR "liver diseases"[Title/Abstract] OR "fatty liver"[MeSH Terms] OR "non alcoholic fatty liver disease"[MeSH Terms] OR "liver failure"[MeSH Terms] OR ("Hepatitis"[MeSH Terms] OR "hepatitis a"[MeSH Terms]) OR "alanine transaminase"[Title/Abstract] OR "aspartate aminotransferases"[Title/Abstract] OR "alkaline phosphatase"[Title/Abstract] OR "gamma-Glutamyltransferase"[Title/Abstract])	988
SCOPUS	((TITLE-ABS-KEY (phospholipid) OR TITLE-ABS-KEY (phosphatidylcholine) OR TITLE-ABS-KEY (phosphatidylethanolamine) OR TITLE-ABS-KEY (phosphatidylinositol) OR TITLE-ABS-KEY (phosphatidylserine) OR TITLE-ABS-KEY (lysophosphatidylcholine))) AND ((TITLE-ABS-KEY ("fatty liver") OR TITLE-ABS-KEY ("non alcoholic fatty liver disease") OR TITLE-ABS-KEY ("liver failure") OR TITLE-ABS-KEY ("liver cirrhosis") OR TITLE-ABS-KEY ("liver disease")))	1,779
Web of Sciences	((((((TS=(liver diseases)) OR TS=(liver cirrhosis)) OR TS=(fatty liver)) OR TS=(non alcoholic fatty liver disease)) OR TS=(hepatitis))) OR TS=(liver failure) AND ((((((TS=(phospholipid)) OR TS=(phosphatidylcholine)) OR TS=(phosphatidylethanolamine)) OR TS=(phosphatidylinositol)) OR TS=(phosphatidylserine)) OR TS=(lysophosphatidylcholine)) OR TS=(lysophosphatidylethanolamine)	6,713
Google Scholar	("phospholipids" OR "phosphatidylcholine" OR "Phosphatidylethanolamines" OR "Phosphatidylinositols" OR "Phosphatidylserines" OR "Lysophosphatidylcholine" OR “Lysophosphatidylethanolamines” OR “lipidomic” OR “metabolomics”) AND ("fatty liver" OR "nonalcoholic fatty liver disease “OR "liver failure" OR "liver cirrhosis" OR "Hepatitis" OR “chronic liver disease”)	2,000
Total		11,480

*Searches were limited to original articles, and studies published in the English language using the appropriate filters and/or search terms depending on the database.
